# SIRT6 mediated histone H3K9ac deacetylation involves myocardial remodelling through regulating myocardial energy metabolism in TAC mice

**DOI:** 10.1111/jcmm.17915

**Published:** 2023-08-21

**Authors:** Shuqi Wu, Jiaojiao Zhang, Chang Peng, Yixiang Ma, Xiaochun Tian

**Affiliations:** ^1^ Department of Pediatrics, Guizhou Children's Hospital Affiliated Hospital of Zunyi Medical University Zunyi Guizhou China

**Keywords:** energy metabolism, histone deacetylation, mechanism, myocardial remodelling, SIRT6

## Abstract

Pathological myocardial remodelling is the initial factor of chronic heart failure (CHF) and is induced by multiple factors. We previously demonstrated that histone acetylation is involved in CHF in transverse aortic constriction (TAC) mice, a model for pressure overload‐induced heart failure. In this study, we investigated whether the histone deacetylase Sirtuin 6 (SIRT6), which mediates deacetylation of histone 3 acetylated at lysine 9 (H3K9ac), is involved pathological myocardial remodelling by regulating myocardial energy metabolism and explored the underlying mechanisms. We generated a TAC mouse model by partial thoracic aortic banding. TAC mice were injected with the SIRT6 agonist MDL‐800 at a dose of 65 mg/kg for 8 weeks. At 4, 8 and 12 weeks after TAC, the level of H3K9ac increased gradually, while the expression of SIRT6 and vascular endothelial growth factor A (VEGFA) decreased gradually. MDL‐800 reversed the effects of SIRT6 on H3K9ac in TAC mice and promoted the expression of VEGFA in the hearts of TAC mice. MDL‐800 also attenuated mitochondria damage and improved mitochondrial respiratory function through upregulating SIRT6 in the hearts of TAC mice. These results revealed a novel mechanism in which SIRT6‐mediated H3K9ac level is involved pathological myocardial remodelling in TAC mice through regulating myocardial energy metabolism. These findings may assist in the development of novel methods for preventing and treating pathological myocardial remodelling.

## 1 BACKGROUND

The incidence of chronic heart failure (CHF) continues to increase worldwide.^1^ To reduce the incidence and clinical and economic burden of patients with CHF, the development of strategies for the prevention of CHF is critical. Studies have confirmed that the key factor in the development of CHF is pathological myocardial remodelling.^2^, ^3^ Pathological myocardial remodelling is a chronic and progressive process; it is characterized by cardiomyocyte hypertrophy and apoptosis, myocardial fibrosis and myocardial energy metabolism disorder.^4^

Myocardial energy metabolism disorder is an important driving factor for pathological myocardial remodelling and occurs at any period of myocardial remodelling.^3^ Reduction of adenosine triphosphate (ATP) production and/or use in the heart results in changes in cardiac metabolic substrate pathways and impaired cardiac systolic function, leading to myocardial remodelling and inevitably CHF.^5^ Therefore, regulating cardiac energy metabolism may be an effective measure to improve cardiac function and alleviate CHF.^6^ As the most active organ in the body, the heart is also one of the organs with the highest mitochondrial content.^7^ During myocardial remodelling, myocardial cell hypertrophy occurs first in the heart, and the number of myocardial cells does not increase but myocardial fibrosis occurs; the structure of energy‐supplying mitochondria is damaged, resulting in a decrease in ATP production. The insufficient energy supply leads to cardiomyocyte apoptosis, which is also one of the important mechanisms of pathological myocardial remodelling.^8^

Angiogenesis is a compensatory mechanism of the cardiac response to hemodynamic pressure elevation.^9^ Studies have found that in the late stage of myocardial remodelling, angiogenesis in myocardial tissue was significantly reduced.^10^, ^11^ Myocardial cell blood supply was insufficient during myocardial remodelling, and hypoxia was aggravated and a large number of myocardial cells died. Heart enlargement and ventricular wall thinning subsequently occurred. Therefore, promoting blood vessel formation may improve myocardial remodelling. However, the underlying mechanisms are still unclear.

Epigenetic modification regulates chromatin structure and specific gene expression in various biological processes and diseases, including during cardiovascular development and disease.^12^ Histone acetylation modification, an epigenetic mechanism, is a reversible modification that regulates the transcriptional activity of genes.^13^ In recent years, research has explored targeting histone acetylation modification for the treatment of pathological myocardial remodelling.^14^, ^15^

Sirtuin 6 (SIRT6) is a member of the III class histone deacetylase (HDAC) Sirtuin family and a highly specific histone H3 deacetylase that targets histone 3 acetylated at Lys‐9 (H3K9), Lys‐56 (H3K56) and Lys‐18 (H3K18).^16^ Mice with heart‐specific knockout of SIRT6 spontaneously develop cardiac hypertrophy and heart failure, while SIRT6 transgenic overexpression blocks pressure overload and agonist‐induced myocardial hypertrophy.^17^, ^18^ SIRT6 has an inhibitory effect on mitochondrial damage in cardiomyocytes,^19^ but the specific mechanism and regulatory targets are still unclear. Increasing evidence has demonstrated that SIRT6 functions as a negative regulator of cardiac hypertrophy.^20^, ^21^ However, the regulatory relationship between SIRT6‐mediated histone deacetylation and the occurrence and development of myocardial remodelling and the specific regulatory mechanism remains unknown.

In the present study, we explored the role and potential mechanism of SIRT6‐mediated histone H3K9ac modification imbalance in myocardial remodelling. We generated a transverse aortic constriction (TAC) mouse model of pressure‐overload myocardial remodelling, and SIRT6‐mediated myocardial energy metabolism was used as an entry point. These findings may provide a new insights into strategies for the prevention and treatment of CHF.

## 2 MATERIALS AND METHODS

### 2.1 Experimental mice

#### 2.1.1 Ethics approval

All procedures were performed in accordance with the Guide for the Care and Use of Laboratory Animals published by the National Institutes of Health. The animal research protocol was approved by the Animal Protection and Use Committee of Zunyi Medical University.

Kunming mice (male, SPF grade, 6–8 weeks, 35 ± 5 g) were purchased from the Animal Center of Zunyi Medical University. Animals were maintained under standard laboratory conditions (25°C, 55%–65% humidity, 12 h light/dark cycle and water ad libitum).

Animals were divided into the following five groups: Normal group, Sham group, TAC + Veh group, TAC group and TAC + MDL‐800 (Yuanye) group (*n* = 6/group). Mice in the TAC groups were deprived of food and drink for 12 h before surgery. To establish the TAC model, animals were intraperitoneally injected with 0.8% sodium pentobarbital (50 mg/kg) for general anaesthesia; the adequacy of surgical anaesthesia was judged by the disappearance of righting reflex and pedal withdrawal reflex. The limbs and paws of mice were fixed on a surgical bed; a 1 cm incision was made close to the sternum after iodophor disinfection, and the two thymus glands were carefully separated to expose the aortic arch. A 4‐0 silk (Jinhuan, China) suture was tied around a blunt part (0.5 mm) of an L‐shaped needle, which was placed contiguous to the aorta between the brachiocephalic trunk and the left carotid artery and removed quickly after the placement of ligation. which could cause about 75% stenosis of the aortic arch. In the Sham group, the aorta was separated without ligation, and the other steps were the same as those in the TAC group. Mice in Normal and Sham group were not treated. In the TAC + MDL‐800 group, 65 mg/kg MDL‐800 (dissolved in DMSO) was administered by intraperitoneal injection three times a week for 12 weeks after 4 weeks of surgery.^22^ The TAC + Veh group received an equal volume DMSO following the same protocol.

### 2.2 Echocardiography measurements

Mice were given 2% isoflurane mixture for anaesthesia and then transferred to the work table for fixation; animals continued to receive 1.5% isoflurane mixture to maintain anaesthesia. We performed transthoracic echocardiograms on mice using a Vevo 2100 High‐Resolution echocardiograph (Visual Sonics), as described previously.^23^ After echocardiography, the left ventricular systolic pressure (LVSP) in mice was obtained by carotid cannulation and measured using the Biomedical Signal Acquisition and Processing System (Taimeng).

### 2.3 Immunofluorescence

Mouse heart tissues were harvested and immediately fixed in 4% paraformaldehyde solution; tissues were fixed at room temperature (RT) overnight, dehydrated and embedded in paraffin the next day. Sections (3 μm) were prepared and dewaxed with xylene and anhydrous ethanol. The tissue sections were incubated in EDTA (pH 8.0) antigen repair solution for antigen repair in a microwave oven. After cooling, the sections were washed with PBS three times for 5 min each, blocked with 3% BSA for 30 min and then incubated with anti‐CD31 monoclonal antibody (1:200, 28083‐1‐AP, Proteintech) at 4°C overnight. Next day, sections were washed with PBS three times for 5 min each. Sections were incubated with Alexa Fluor 594 goat anti‐rabbit IgG H & L (1:300, HA1117, HAUBIO) at RT for 1 h. Samples were washed in PBS; then, the sections were stained with DAPI at RT in dark for 10 min. The sections were sealed with anti‐fluorescence quenching sealing agent and observed under a fluorescence inverted microscope. Images were evaluated using Image Pro Plus professional image analysis software for quantitative analysis.

Microvessel density (MVD) was defined as the average count of microvessels composed of a single or a group of ECs with positive for CD31 staining. First scan the whole section under low light microscope to look for three high density areas, the number of vessels with positive staining was then counted under 200× microscope. By using Image‐pro Plus 6.0 (Media Cybernetics) software for computerized analysis, the average number of blood vessels in the three fields of view was calculated as MVD value (n/HP).

### 2.4 Masson's staining

Sections were taken from each group for dewaxed, then hydrated in different concentrations of ethanol solution and soaked in double steaming water for 5 min. The sections were placed in Bouin solution and after media stained overnight at room temperature, the distilled water was rinsed until the yellow disappeared on the sections. According to the instructions of the modified Massone tricholor staining kit (BioBomei), reagents were added successively for staining, the stained sections were washed with different concentrations of ethanol solution, and the last xylene was transparent for three times before the neutral resin sealing microscopy.

### 2.5 Western blotting (WB)

Nucleoproteins were extracted from mouse myocardial tissues using a Minute™ kit (Invent) following the kit instructions. The nucleoprotein extracts were separated on SDS‐PAGE gels and electro‐transferred onto polyvinylidene difluoride (PVDF) membranes (pore size 0.45/0.22 μm, Merck Millipore Ltd.). The membranes were then blocked with 5% skim milk at RT for 1 h, washed in TBST three times 10 min each and then incubated overnight at 4°C with specific primary antibodies (anti‐SIRT6 [1:2000, abs110527, Absin], anti‐H3K9ac [1:1000, abs145132, Absin], anti‐VEGFA [1:2000, 19003‐1‐AP, Proteintech], anti‐H3 [1:2000, 17168‐1‐AP, Proteintech] and anti‐GAPDH [1:2000, 10494‐1‐AP, Proteintech]). The membranes were washed three times with TBST for 10 min and then incubated with secondary antibodies (anti‐rabbit IgG [1:5000, SA00001‐2, Proteintech], anti‐mouse IgG [1:5000, SA00001‐1, Proteintech] at RT for 1 h. After three washes in TBST for 10 min, bands on the immunoblots were visualized by enhanced chemiluminescence (Thermo Fisher Scientific). Immunopositive signals were then scanned, and bands were quantified using the Quantity One (Version 4.4) software package (Bio‐Rad).

### 2.6 Co‐immunoprecipitation (CoIP)

CoIP experiments were performed using the co‐immunoprecipitation kit (Beaver) in accordance with the manufacturer's instructions. Myocardial tissues were washed in PBS twice, and the solution was discarded. The sample was mixed with binding buffer and phenylmethylsulfonyl fluoride (PMSF) with a final concentration of 1 mM and ground using a high‐throughput grinder, followed by 10 min standing on ice before centrifugation (4°C, 12,000 *g*, 10 min). The supernatant was collected. Protein A/G beads (50 μL) were washed with 200 μL binding buffer solution for 2 min; magnetic separation was performed for 30 s, the supernatant was discarded and antibody working solution (200 μL) was added (anti‐H3K9ac [1:100, abs145132, Absin], anti‐IgG (1:250, B900620, Proteintech). The mixture was incubated at RT for 30 min; magnetic separation was performed for 30 s, the supernatant was discarded and the protein A/G bead/antibody complex was washed. Then, 200 μL myocardial tissue supernatant was added. The mixture was incubated at 4°C for 12 h. The next day, the protein A/G bead/antibody/antigen complex was magnetically separated for 30 s, washed with 200 μL washing buffer for 2 min and magnetically separated for 30 s twice. The magnetic beads/antibody/antigen complex was transferred to a new EP tube for magnetic separation, and the supernatant was discarded. The sample was mixed with 1 × SDS‐PAGE Loading Buffer and heated at 95°C for 5 min. After centrifugation (RT, 12,000 *g*, 10 min), the supernatant was collected for WB.

### 2.7 Transmission electron microscopy (TEM)

Fresh mouse heart tissues were harvested and immediately placed in a petri dish containing electron microscope fixative. The tissues were cut into 1 mm^3^ tissue blocks with a autoclaved blade, transferred to an EP tube with fresh electron microscope fixative and fixed at 4°C for 2 h. After washing with phosphate buffer, the samples were dehydrated in graded ethanol and then embedded with acetone and EMBed 812. Ultra‐thin sections (70 nm thick) were cut by ultra‐thin slicing machine and then stained with 2% uranium acetate saturated alcohol solution and 2.6% lead citrate. Samples were analysed on a transmission electron microscope (Hitachi). Mitochondrial size (μm), area (μm^2^) and mitochondrial density per unit area (44.32543653923 μm^2^) were measured by Image‐Pro Plus 6.0 software with 7k 2 μm scale as the standard.

### 2.8 Mitochondrial respiratory function detection

Myocardial mitochondria were extracted using the Biyuntian Tissue Mitochondria Isolation Kit (Biyuntian), following the manufacturer's instructions. Bradford method was used to quantify extracted mitochondria. Subsequently, the oxidative phosphorylation (OXPHOS) capacity of isolated mitochondria was detected by Oxygraph‐2K (Oroboros) following the manufacturer's instructions. Turn on the power supply, clean the A and B warehouses three times each, then A and B warehouses each add 2.5 mL Miro5 (EGTA 0.5 mM, MgCl_2_·6H_2_O 3 mM, lactobionic acid 60 mM, taurine 20 mM, KH_2_PO_4_ 10 mM, HEPES 20 mM, sucrose 110 mM, BSA 1 g/L, pH 7.1). The cover was closed so that the two reaction chambers are in a closed state. After the balance of oxygen value and oxygen consumption value, the calibration was carried out, and the reaction substrate was gradually added in the following order: 100 μg mitochondria, followed by the addition of complex I substrate malate (0.8 M, 5 μL), glutamate (2 M, 10 μL) and ADP (0.5 M, 4 μL); the oxidative phosphorylation ability of mitochondrial complex I was recorded. Finally, succinate (2 M, 10 μL) was added to record the maximum oxidative phosphorylation ability of coupled complexes I and II. After all the reactions were completed, data were obtained and analysed.

### 2.9 Statistical analysis

All data are expressed as mean ± standard deviation (SD). All statistical analyses were performed using the SPSS statistical software package version 18.0 (SPSS Inc.). Data were compared using the least significant differences (LSD)‐*t* test and by one‐way analysis of variance (anova) after testing raw data for normality first. *p*‐Values <0.05 were statistically significant.

## 3 RESULTS

### 3.1 Validation of the model and myocardial remodelling in TAC mice

**FIGURE 1:**
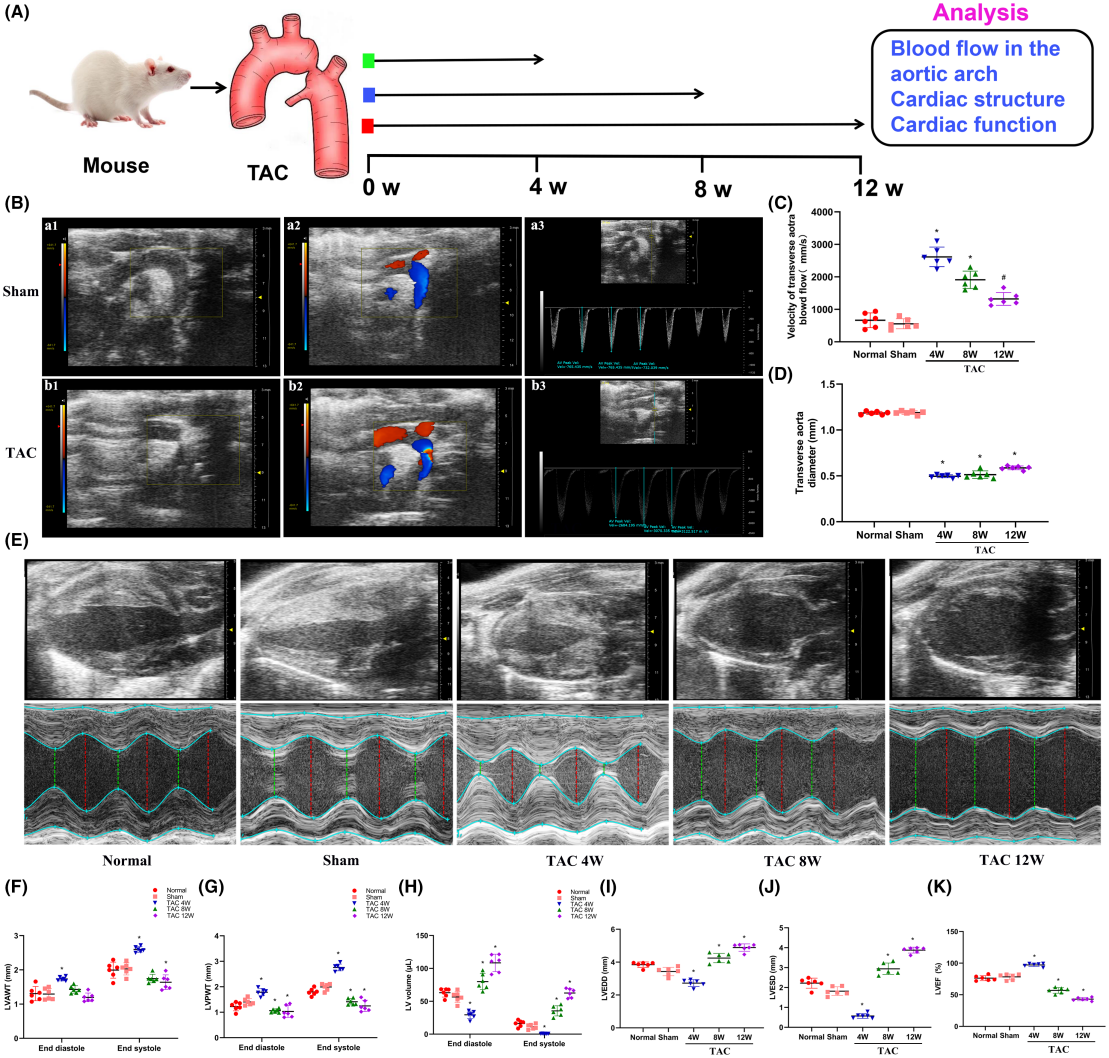
The changes of cardiac structure and function in thoracic aortic constriction (TAC) mice during myocardial remodelling. (A) Schematic of the experimental plan to study the cardiac structure and function in TAC mice. (B) Echocardiography imaging of the aortic arch in mice. a1 and b1: ultrasound graph of the aortic arch in the Sham group and TAC group, the aortic arch was significantly narrowed in TAC group (red arrow); a2 and b2: colour Doppler imaging in the aortic arch showed smooth blood flow in the sham group and blocked blood flow in TAC mice; a3 and b3: Pulse Doppler ultrasound was used to measure the velocity of blood flow in the aortic arch in the Sham group and the TAC group. (C) Statistical analysis of the velocity of transverse aorta blood flow in each group. (D) Statistical analysis of the diameter at transverse aortic ligation in each group. (E) Cardiac ultrasound of mice in each group; red or green dashed lines indicate the diameter of the left ventricle during diastole or systole. (F–H) Statistical analysis of LVAWT, LVPWT and LVV in left ventricular end‐systolic and end‐diastolic. (I, J) Statistical analysis of LVEDD and LVESD. (K) Statistical analysis of LVEF. LVAWT, left ventricular anterior wall thickness; LVEDD, left ventricular end‐diastolic dimension; LVEF, left ventricular ejection fraction; LVESD, left ventricular end‐systolic dimension; LVPWT, left ventricular posterior wall thickness; LVV, left ventricular volume; TAC, transverse aortic constriction; W, week. *p < 0.05 vs. the Sham group, *n* = 6.

We created a mouse model of TAC by the application of thoracic aortic banding (TAB). We then used echocardiography to evaluate the effects of TAB and to assay cardiac structure and function in mice at 4, 8 and 12 weeks after TAB (Figure 1A). Echocardiography data showed that the ligation site between the innominate artery, the left carotid artery and the ligated aortic arch was clearly constricted (Figure 1B‐b1). Colour Doppler flow imaging (CDFI) further showed that blood flow was blocked in the aortic arch (Figure 1B‐b2), and velocity increased in the aortic arch (Figure 1B‐b3). No changes in the above indicators were observed in the Sham group (Figure 1B‐a1–a3). The velocity of transverse aorta blood flow in TAC mice at 4 and 8 weeks after TAB was higher than that of the Sham group (Figure 1C), and the diameter of the aortic arch ligation was significantly reduced in the TAC group compared with the Sham group (Figure 1D). These data showed that the TAC mouse model was successfully established.

Pathological myocardial remodelling is a necessary stage for myocardial hypertrophy to transition into CHF. Next, the cardiac structure and function in TAC mice at 4, 8 and 12 weeks after TAB were examined by echocardiography (Figure 1E). Echocardiography showed that the left ventricular (LV) wall in TAC mice had gradually thickened from 4 to 8 weeks after TAB compared with observations in the Sham group. Notably, at 8 to 12 weeks after TAB, the LVAWT and LVPWT of TAC mice began to gradually decrease (Figure 1F,G), while the LVV of TAC mice began to gradually increase (Figure 1H). The LVEDD of TAC mice at 4 weeks after TAB was significantly decreased compared with that of the Sham group (*p* < 0.05), while the LVEDD of TAC mice at 12 weeks after TAB was significantly increased (*p* < 0.05) (Figure 1I). The LVESD of TAC mice at 4 weeks after TAB was significantly decreased compared with that of the Sham group (*p* < 0.05), while the LVESD of TAC mice at 8 and 12 weeks after TAB was significantly increased (*p* < 0.05) (Figure 1J). The LVEF of TAC mice at 4 weeks after TAB was significantly increased compared with that of the Sham group (*p* < 0.05), while the LVEF of TAC mice at 8 and 12 weeks after TAB was significantly decreased (*p* < 0.05) (Figure 1K).

### 3.2 The levels of VEGFA, SIRT6 and H3K9ac in myocardial tissues of TAC mice

**FIGURE 2:**
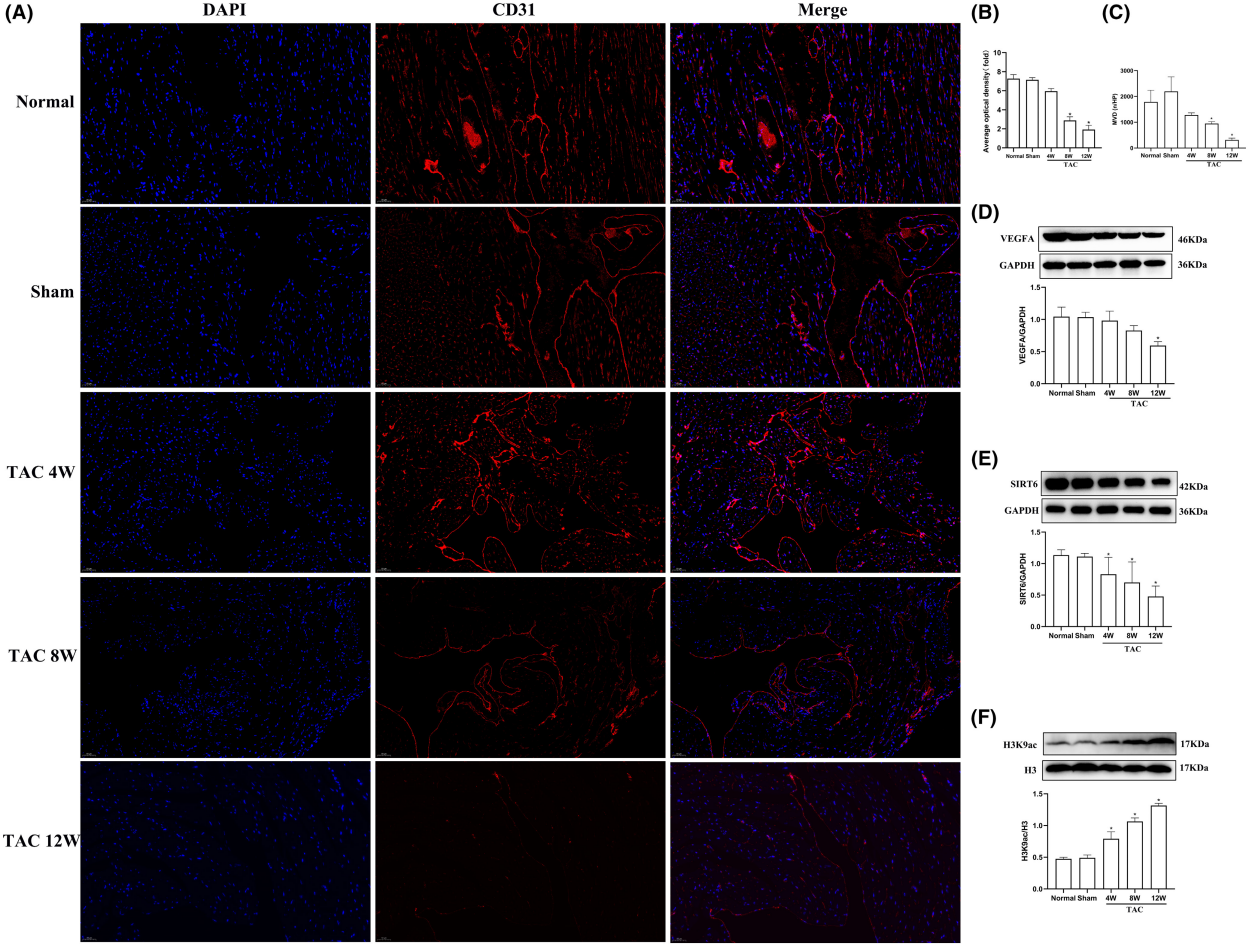
Levels of SIRT6, VEGFA, and H3K9ac and angiogenesis in myocardial tissues of TAC mice. (A) CD31 expression in myocardial cells was detected by immunofluorescence. Red fluorescence indicates CD31, and blue fluorescence indicate nuclei (DAPI stain). Scale bar = 50 μm. (B) Average optical density in each group. (C) Quantitative statistics of the MVD (*n* = 3). (D–F) Protein expression levels of VEGFA, SIRT6 and H3K9ac were examined by western blotting (*n* = 6). H3K9ac, acetylated lysine 9 on histone H3; TAC, thoracic aortic constriction; W, weeks. *p < 0.05 vs. the Sham group.

Studies reported that histone deacetylation mediated by SIRT6 may participate in myocardial remodelling induced by pressure overload, and the angiogenesis in myocardial tissues was reduced during myocardial remodelling.^24^ Next, the levels of VEGFA, SIRT6 and H3K9ac in myocardial tissues of TAC mice were examined. In our report, immunofluorescent staining showed that the expression of the vascular endothelial adhesion factor CD31 and MVD in TAC mice at 8 and 12 weeks after TAB decreased significantly compared with that of the Sham group (*p* < 0.05) (Figure 2A–C), which represents a decrease in angiogenesis. WB also showed that the expression of VEGFA in TAC mice at 12 weeks after TAB was significantly decreased compared with that of the Sham group (*p* < 0.05) (Figure 2D). In addition, the protein levels of SIRT6 in TAC mice at 4, 8 and 12 weeks after TAB were lower than those in the Sham group (*p* < 0.05) (Figure 2E), while H3K9ac level was increased significantly in the same samples (*p* < 0.05) (Figure 2F). This suggests that hyperacetylation of H3K9ac mediated by SIRT6 may be involved in myocardial remodelling induced by pressure‐overload in TAC mice.

### 3.3 The SIRT6 agonist MDL‐800 attenuates H3K9ac hyperacetylation and promotes angiogenesis in TAC mice

**FIGURE 3:**
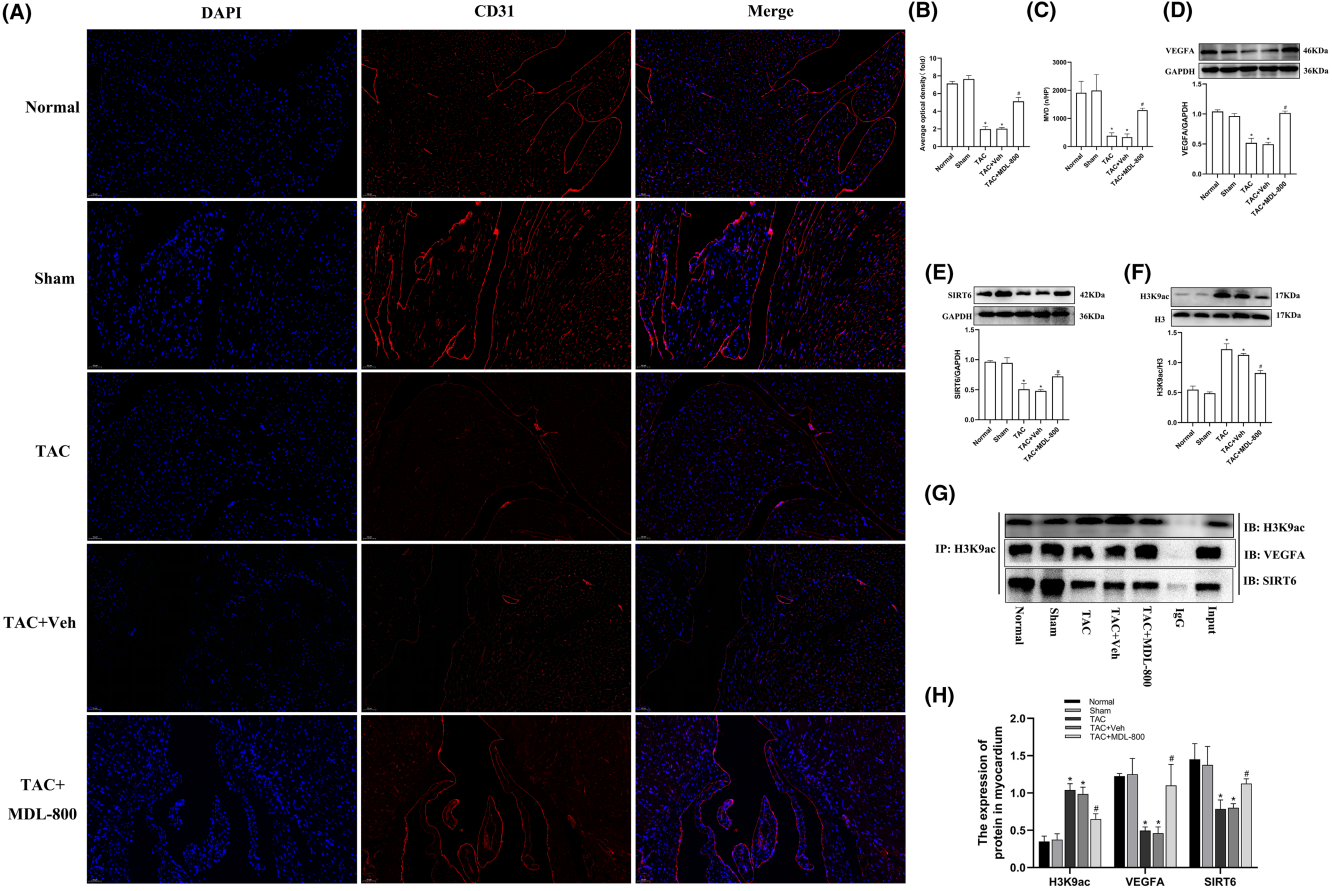
The SIRT6 agonist MDL‐800 promotes VEGFA expression and myocardial angiogenesis by deacetylating H3K9ac. (A) CD31 expression in myocardial cells was detected by immunofluorescence. Red fluorescence indicates CD31, and blue fluorescence indicate nuclei (DAPI stain). Scale bar = 50 μm. (B) Average optical density in each group. (C) Quantitative statistics of the MVD (*n* = 3). (D–F) Protein expression levels of VEGFA, SIRT6 and H3K9ac were assayed by western blotting (*n* = 6). (G) CoIP of myocardial tissue lysates under five different experimental conditions using anti‐H3K9ac protein G magnetic beads. Immunoblot (IB) for SIRT6 and VEGFA was performed. (H) Quantification analysis of CoIP results (*n* = 3). TAC, transverse aortic constriction; MDL‐800, SIRT6 agonist; Veh, vehicle; H3K9ac, acetylated lysine 9 on histone H3; Input, positive control; IgG, negative control; IP, immunoprecipitation; IB, immunoblotting. *p < 0.05 vs. the Sham group, #*p* < 0.05 vs. the TAC group.

To evaluate whether SIRT6‐mediated H3K9ac hyperacetylation is involved in the regulation of VEGFA, TAC mice were treated by the SIRT6 agonist MDL‐800 from 4 to 12 weeks after TAB, and the hearts were collected at 12 weeks for analysis. Immunofluorescent staining showed that the expression of the vascular endothelial adhesion factor CD31 and the MVD in the TAC group was significantly decreased compared with that of the Sham group (*p* < 0.05), while MDL‐800 improved the reduction of CD31 (Figure 3A–C). WB showed that VEGFA expression in the TAC group was significantly decreased compared with that in the Sham group (*p* < 0.05), while MDL‐800 improved the reduction of VEGFA (Figure 3D). While SIRT6 expression was decreased significantly in the TAC group compared with the Sham group (*p* < 0.05), MDL‐800 treatment resulted in increased expression (Figure 3E). H3K9ac level in the hearts of the TAC group was increased compared with levels in the Sham group (*p* < 0.05), while MDL‐800 reduced H3K9ac levels (Figure 3F). CoIP experiments were conducted to examine the formation of a complex between SIRT6 mediated‐H3K9ac and VEGFA. The results demonstrated that SIRT6 interacts with H3K9ac and regulates the gene expression levels of VEGFA (Figure 3G,H).

### 3.4 The SIRT6 agonist MDL‐800 improves cardiac function and LVSP in TAC mice

**FIGURE 4:**
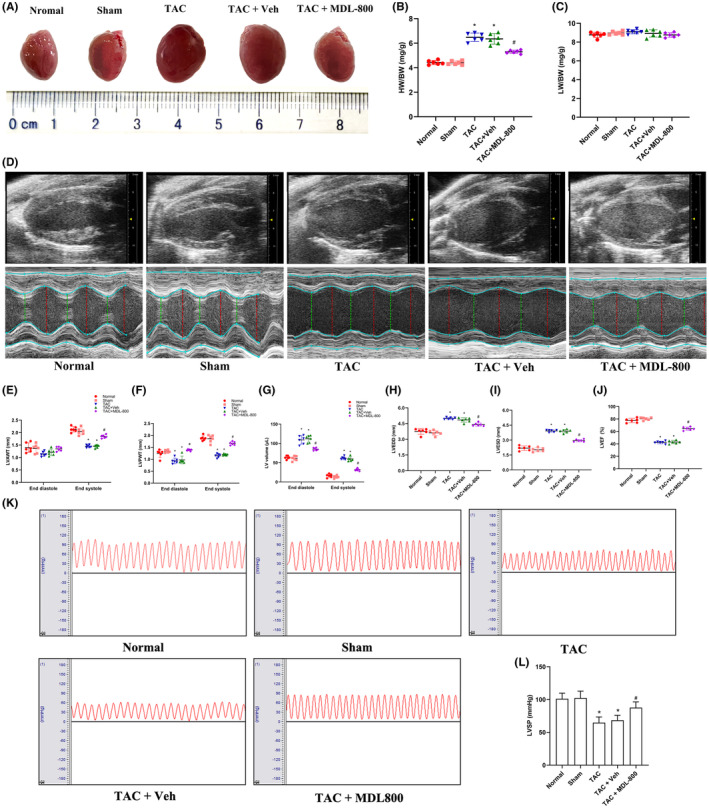
MDL‐800 improves cardiac structure and function in TAC mice. (A) Stereoscopy images of complete hearts in each group. (B) Statistics of cardiac index in mice. (C) Statistics of lung index in mice. (D) Cardiac echocardiography imaging in each group of mice; red or green dotted lines represent the diameters of the left ventricle at diastolic or systolic stages. (E–G) Statistical analysis of LVAWT, LVPWT and LVV at the end of left ventricular diastole and the end of left ventricular systole. (H, I) Statistical analysis of LVEDD and LVESD. (J) Statistical analysis of LVEF. (K) The LVSP of the mice in each group. (L) Statistical analysis of LVSP. BW, body weight; HW, heart weight; LVAWT, left ventricular anterior wall thickness; LVEDD, left ventricular end‐diastolic dimension; LVEF, left ventricular ejection fraction; LVESD, left ventricular end‐systolic dimension; LVPWT, left ventricular posterior wall thickness; LVV, left ventricular volume; LW, lung weight; MDL‐800, SIRT6 agonist; TAC, transverse aortic constriction; Veh, vehicle. *p < 0.05 vs. the Sham group, *#p* < 0.05 vs. the TAC group, *n* = 6.

Pathological myocardial remodelling involves changes in myocardial structure and function. Stereoscopy data showed that the hearts of TAC mice were enlarged compared with the hearts of the Sham group, while the SIRT6 agonist MDL‐800 reversed heart enlargement of TAC mice (Figure 4A). The cardiac index (CMI) of the TAC group was higher than that in the Sham group (*p* < 0.05). However, there was no statistical difference in lung index (LMI) (*p* > 0.05). The SIRT6 agonist MDL‐800 significantly reduced CMI in TAC mice (*p* < 0.05), but LMI remained unchanged in the presence of MDL‐800 (Figure 4B,C).

Next, cardiac structure and function in TAC mice were tested by echocardiography (Figure 4D). Echocardiography showed that LVAWT in TAC mice at end‐systole was significantly decreased compared with that in the Sham group (*p* < 0.05), and the SIRT6 agonist MDL‐800 increased LVAWT in the hearts of TAC mice, but the LVAWT at end‐diastole was unchanged in the same samples (Figure 4E). LVPWT in TAC mice at end‐systole and end‐diastole was significantly decreased compared with that in the Sham group (*p* < 0.05), and the SIRT6 agonist MDL‐800 increased LVPWT (Figure 4F). The LVV in TAC mice at end‐systole and end‐diastole were increased compared with that of the Sham group (*p* < 0.05), and the SIRT6 agonist MDL‐800 reversed the LVV increase (Figure 4G). LVEDD and LVESD in the hearts of TAC mice were increased significantly compared with that in the Sham group (*p* < 0.05), and the SIRT6 agonist MDL‐800 attenuated the LVEDD and LVESD increase (Figure 4H,I). LVEF was significantly decreased in the TAC group compared with that in the Sham group (*p* < 0.05), and MDL‐800 improved LVEF (Figure 4J). In addition, LVSP was significantly reduced in the TAC group compared with that in the Sham group (*p* < 0.05), while MDL‐800 improved LVSP in the hearts of TAC mice (Figure 4K,L). These data implied that the SIRT6 agonist MDL‐800 improved cardiac structure and function in TAC mice.

### 3.5 The SIRT6 agonist MDL‐800 alleviates mitochondria damage and myocardial fibrosis in the hearts of TAC mice

**FIGURE 5:**
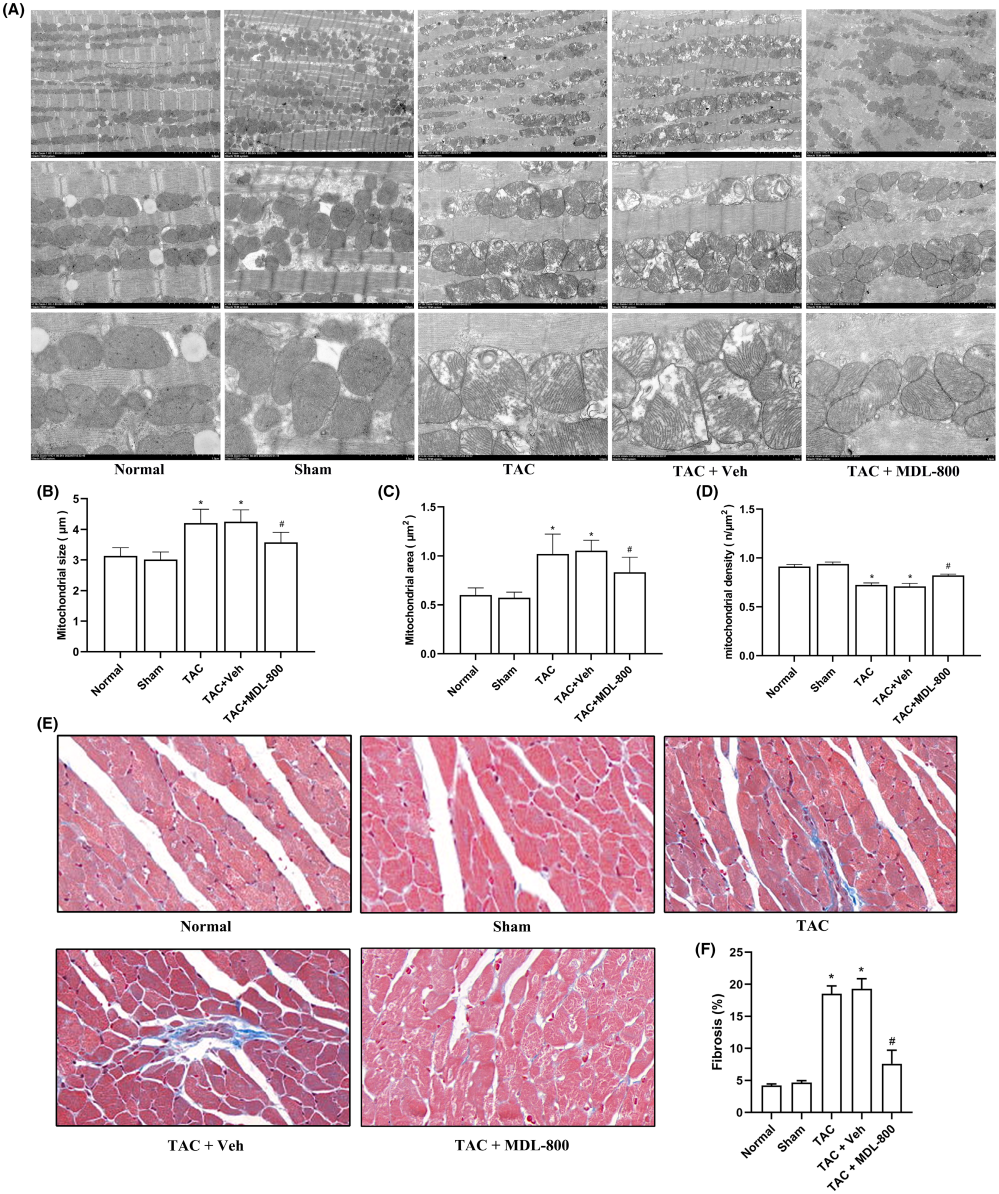
MDL‐800 improved myocardial mitochondrion ultrastructure in TAC mice. (A) The ultrastructure of left ventricle of mice in each group was examined by transmission electron microscopy (TEM). Magnification, ×2500, ×7000, ×15,000. (B) Statistics of mitochondrial size. (C) Statistics of mitochondrial area. (D) Statistics of mitochondrial density. (E) Sections of heart tissues treated with Masson's stain (Magnification, ×20). (F) Statistical analysis of myocardial fibrosis. MDL‐800, SIRT6 agonist; TAC, transverse aortic constriction; Veh, vehicle. *p < 0.05 vs. the Sham group, *#p* < 0.05 vs. the TAC group, *n* = 3.

The mitochondrion is the energy factory of myocardial cells, and damage to mitochondria often leads to insufficient energy supply of myocardial tissues, which is closely related to CHF. To assess myocardial injury at the microstructure level, the ultrastructure of myocardial cells was observed using TEM. As shown in Figure 5A, no obvious microstructural changes and damages were found in the Sham group and Normal group. The TAC group cardiomyocytes showed severe myofilament rupture, swollen mitochondria, mitochondrial cristae damage or disappearance and mitochondrial vacuolation, and the SIRT6 agonist MDL‐800 attenuated mitochondrial ultrastructure damage. The size and area of mitochondria in the TAC group were significantly increased compared with that in the Normal group and Sham group (*p* < 0.05) (Figure 5B,C), confirming mitochondrial damage, while the mitochondrial density was significantly reduced (*p* < 0.05) (Figure 5D). However, in the TAC + MDL‐800 group, the size, area and density of mitochondria were significantly improved. In addition, we further examined the condition of myocardial fibrosis in the hearts of mice, and found that the myocardial fibrosis was evident in the TAC group compared with the Sham group (*p* < 0.05), while the myocardial fibrosis was significantly alleviated in the TAC + MDL‐800 group (*p* < 0.05) (Figure 5E,F).

### 3.6 The SIRT6 agonist MDL‐800 improves mitochondrial respiratory function in the hearts of TAC mice

**FIGURE 6:**
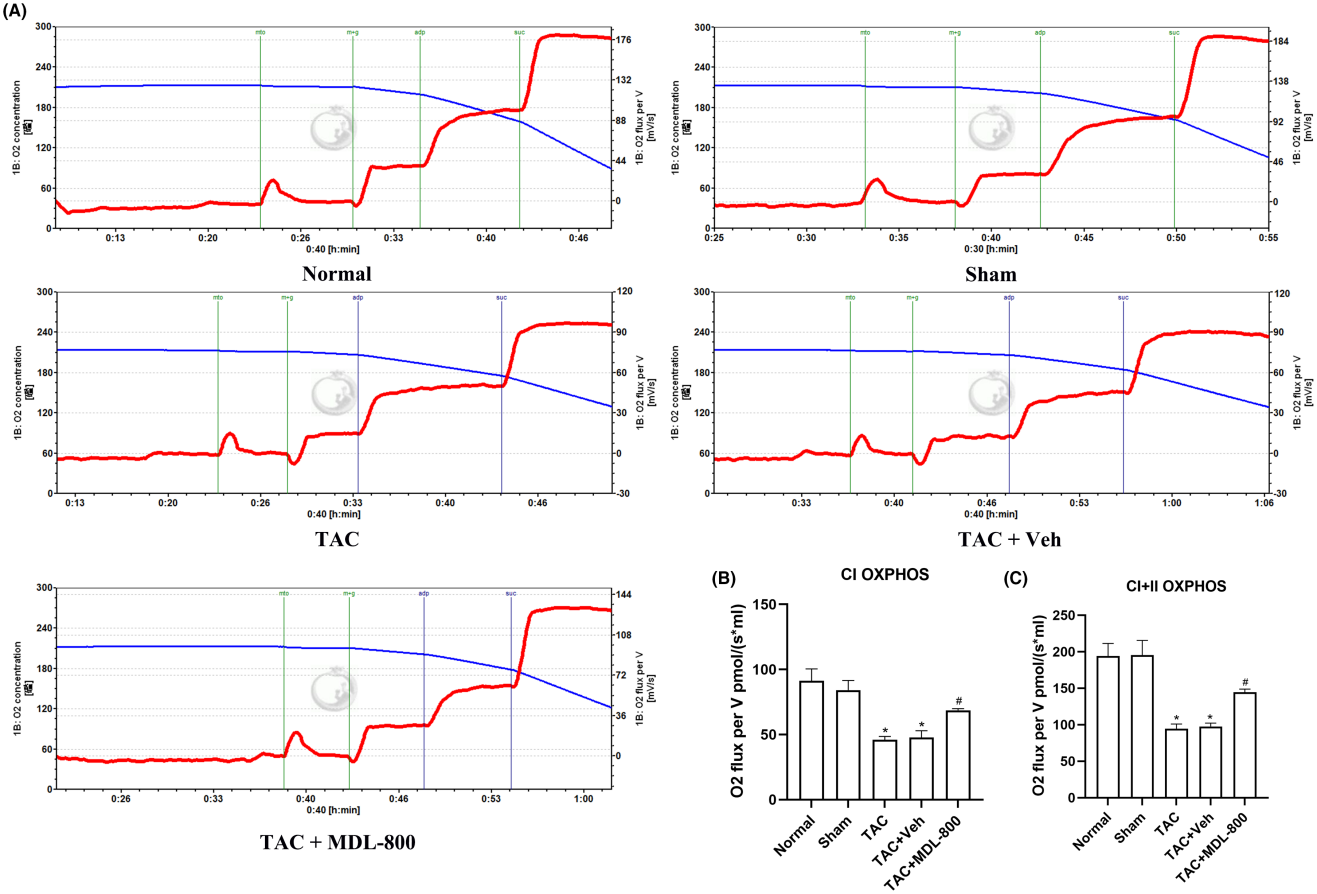
MDL‐800 improves mitochondrial respiratory function. (A) Mitochondrial respiration was measured by high‐resolution respirometry (Oxygraph‐2K). (B, C) Quantitative analysis of the oxidative phosphorylation (OXPHOS) levels of mitochondrial complex I (CI) and the maximum oxidative phosphorylation capacity of the coupled complex I and II (CI + II). The red line indicates the oxygen consumption rate, and the blue line indicates the oxygen concentration in the warehouse. CI, complex I; CI + II, complex I and II; TAC: transverse aortic constriction; MDL‐800, SIRT6 agonist; Veh, vehicle. *p < 0.05 vs. the Sham group, *#p* < 0.05 vs. the TAC group, *n* = 5.

The respiratory function of mitochondria was assessed by measuring the oxygen consumption of myocardial mitochondrial complexes using the Oxygraph‐2k mitochondrial respiratory function apparatus (Figure 6A). The respiratory function of mitochondria in the TAC group was significantly suppressed compared with the Sham group (*p* < 0.05), while the SIRT6 agonist MDL‐800 improved the mitochondrial respiratory function (Figure 6B,C).

## 4 DISCUSSION

Pathological myocardial remodelling is a process of cardiac function conversion from compensation to decompensation in patients with various cardiovascular diseases and is one of the most important pathological mechanisms of CHF.^25^ Pathological myocardial remodelling involves a change in myocardial structure, function and phenotype caused by a series of complex molecular mechanisms; its pathogenesis is complex, and it is currently considered to be a polygenic disease caused by genetic and environmental factors.^2^ The factors affecting pathological myocardial remodelling include ischemia and hypoxia, mechanical stretch, pressure overload, cardiomyopathy and vasoactive substances.^26^ In recent years, delaying myocardial remodelling has become the consensus of clinical treatment of cardiovascular diseases,^27^ and new therapeutic targets are urgently needed.

Epigenetics serves as a link between genes and the environment.^28^ Histone acetylation is one of the mechanisms of epigenetic regulation. The acetylation status of histones determines the transcriptional activity of genes. Histone acetylation regulates transcription by weakening the electrostatic interaction between DNA and histones and between adjacent nucleosomes.^29^ Studies have shown that myocardial remodelling, which is regulated by the activation and inhibition of various factors, involves multiple acetylation regulation mechanisms.^30^

The class III histone deacetylase Sirtuins family is a homologue of the Sir2 family of NAD+ dependent histone deacetylases. Seven sirtuins, SIRT1 to SIRT7, have been identified thus far.^31^ SIRT6 is located in the nucleus and regulates various biological processes such as DNA repair, gene expression and telomere maintenance.^32^ SIRT6 has been shown to regulate various signalling pathways such as myocardial hypertrophy, inflammatory injury and myocardial remodelling.^33^ SIRT6 is a H3K9ac deacetylase, and our previous study showed that H3K9ac acetylation levels increased in mouse cardiac hypertrophy.^34^ Studies have confirmed that H3K9ac acetylation levels are increased in animal models of myocardial remodelling after myocardial infarction and diabetic myocardial damage.^35^, ^36^ Based on these findings, we speculated that the molecular mechanism of SIRT6 in the regulation of pathological myocardial remodelling may be related to the regulation of H3K9ac acetylation mediated by SIRT6. Therefore, we explored the dynamic expression and role of SIRT6 mediated‐H3K9 deacetylation in myocardial remodelling by constructing a pressure‐overload myocardial remodelling mouse model. Our results showed that the expression of SIRT6 was significantly reduced during myocardial remodelling, and the gradual reduction of SIRT6 was accompanied by a continuous remodelling of cardiac structure and progressive decline of cardiac function. Furthermore, the change of histone H3K9ac level was opposite to SIRT6 level. The level of H3K9ac acetylation significantly increased when compensatory cardiac hypertrophy occurs and remained high while the cardiac function is reconstructed and deteriorated. Noteworthily, the data of CoIP also confirmed the interactive regulation between SIRT6 and H3K9ac in the hearts of TAC mice, but full quantitative analysis between them was not performed. Through these findings, we confirmed that the molecular mechanism of SIRT6 in regulating pathological myocardial remodelling may be associated with its function in regulating the hyperacetylation of histone H3K9ac.

In recent years, the strategy of regulating myocardial energy metabolism to treat myocardial remodelling has been widely discussed.^37^, ^38^ Our previous studies demonstrated that histone acetylation regulation is involved in the development of myocardial hypertrophy and myocardial fibrosis during pathological myocardial remodelling.^34^, ^39^ Myocardial energy metabolism disorders are closely related to myocardial remodelling and significantly aggravate myocardial remodelling processes.^40^ Whether histone acetylation modification is involved in regulating myocardial energy metabolism during myocardial remodelling has not been clear. In this study, we confirmed by TEM that the mitochondrial structure was damaged during myocardial remodelling in TAC mice, and the mitochondrial cristae were disordered and even vacuolated. By Oxygraph‐2k mitochondrial respiratory function measurement, we found that the oxidative phosphorylation ability of myocardial mitochondria in TAC mice was decreased and the mitochondrial respiratory function was significantly impaired. The heart is the most energy‐consuming organ in the human body. Myocardial energy metabolism disorder is caused by an imbalance of ATP generation or decomposition in the heart. More than 95% of ATP in the heart is produced by mitochondrial oxidative phosphorylation, and less than 5% of ATP is produced by glycolysis.^41^ Any minor changes in ATP production and metabolism in myocardial tissues will affect the normal function of the heart. Mitochondria are the centre of myocardial energy metabolism, and their structural integrity and normal function are the basis for normal cardiac electrophysiological activity.^42^ Studies have reported that mitochondrial damage can aggravate cardiac dysfunction caused by pressure overload.^43^, ^44^ SIRT6 also plays a crucial role in mitochondrial oxidative regulation and glucose and lipid metabolism.^45^ Activation of SIRT6 enhances mitophagy to exert cardioprotective effects.^46^ SIRT6 was shown to be involved in the regulation of myocardial energy metabolism after ischemia–reperfusion injury by regulating gene expression of the *Nrf2* transcription factor.^21^ However, the deacetylation regulation mechanism of SIRT6 in improving energy metabolism disorders in myocardial remodelling has remained unknown.

Angiogenesis improves the delivery of oxygen and nutrients in myocardial tissues. Mitochondria coordinate changes in energy metabolism by sensing oxygen in tissues and produce reactive oxygen species to maintain the internal environment of the organism.^47^ Promoting angiogenesis improves myocardial energy metabolism disorders and mitochondrial damage by enhancing oxygen delivery to hypoxic tissues. Vascular endothelial growth factor A (VEGFA), a member of the VEGF family, is a potent angiogenic factor.^48^ In animal models of ventricular remodelling after acute myocardial infarction, up‐regulation of VEGFA expression reduces left ventricular remodelling and improves cardiac function.^49^ Increased expression of angiogenic factor VEGFA also improves microvascular formation and oxygen diffusion, thereby alleviating cardiac fibrosis and myocardial remodelling.^50^ Studies have also demonstrated that angiogenesis is regulated by histone acetylation modification.^51^, ^52^ Histone acetylase P300 regulates the transcription of the VEGFA gene,^51^ but the specific pathway and regulatory targets are still unclear. In this study, we examined the expression of VEGFA and angiogenesis in myocardial tissues during pathological myocardial remodelling. The results showed that the expression of VEGFA was significantly decreased in the decompensated stage of myocardial remodelling, and immunofluorescence also showed that the expression of CD31 was significantly decreased. CD31 is a marker of angiogenesis^53^; the reduction of both CD31 and MVD confirmed the capillary rarefaction in myocardial tissues. Based on the above, we hypothesized that mitochondrial damage caused by the reduced blood oxygen pathway may be a regulatory target for myocardial remodelling in TAC mice caused by hyperacetylation of H3K9ac. Therefore, CoIP experiment was used again to confirm the interactive regulation between VEGFA and H3K9ac. We further used MDL‐800 to induce SIRT6 in myocardial tissues and down‐regulate the level of H3K9ac. We found that structural remodelling of the heart was improved by downregulating the level of H3K9ac in the myocardium of TAC mice; the low expression of VEGFA in myocardium was improved, and MVD in myocardium was increased. TEM also confirmed that down‐regulation of H3K9ac acetylation level significantly improved the structural damage of myocardial mitochondria; meanwhile, the myocardial fibrosis was also ameliorated. Oxygraph‐2k mitochondrial respiratory function test also confirmed that mitochondrial complex I and mitochondrial maximum oxidative phosphorylation increased after down‐regulation of H3K9ac acetylation level, which significantly improved mitochondrial respiratory function.

**FIGURE 7:**
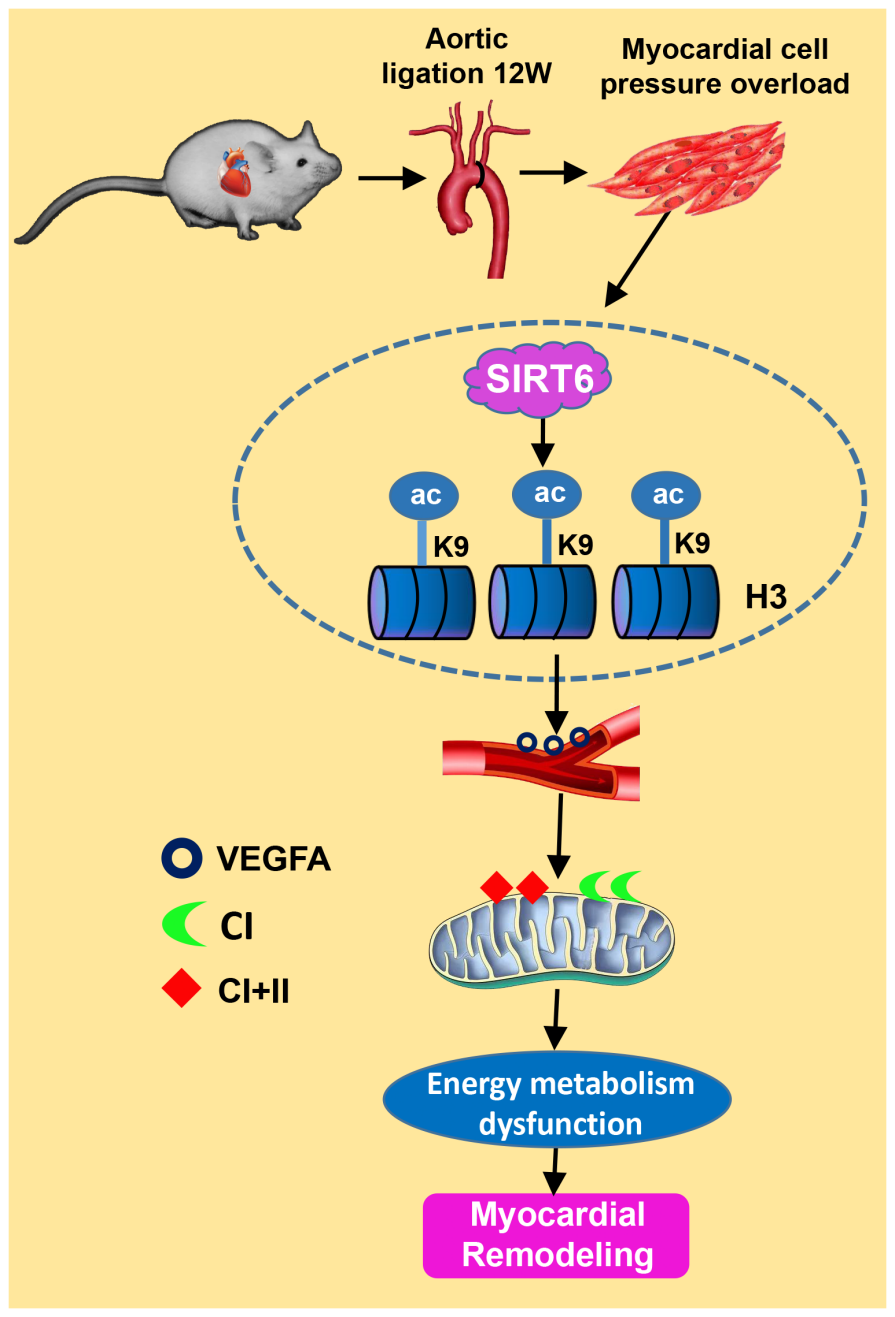
Schematic representation of the potential mechanism by which SIRT6 regulates myocardial energy metabolism to attenuate myocardial remodelling in TAC mice. Histone deacetylase SIRT6 regulates VEGFA expression by deacetylating H3K9ac to improve myocardial energy metabolism and delay pathological myocardial remodelling in TAC mice.

In Figure 7, we show the proposed mechanism by which SIRT6 regulates myocardial energy metabolism to attenuate myocardial remodelling in TAC mice. Overexpression of SIRT6 down‐regulated the acetylation level of H3K9ac, promoted myocardial angiogenesis in the process of myocardial remodelling, improved the delivery of nutrients such as oxygen, and ameliorated mitochondrial structure and function damage, so that myocardial energy metabolism disorders can be restored, and myocardial remodelling was significantly improved. The results of this study may provide a new potential treatment strategy for clinical prevention and treatment of myocardial remodelling caused by pressure overload.

## AUTHOR CONTRIBUTIONS

**Shuqi Wu:** Conceptualization (equal); data curation (equal); methodology (equal); validation (equal); writing – original draft (equal); writing – review and editing (equal). **Jiaojiao Zhang:** Formal analysis (equal); investigation (equal). **Chang Peng:** Conceptualization (equal); funding acquisition (equal); methodology (equal); writing – original draft (equal); writing – review and editing (equal). **Yixiang Ma:** Investigation (equal); resources (equal). **Xiaochun Tian:** Investigation (equal); resources (equal).

## FUNDING INFORMATION

This study was supported by the National Natural Science Foundation of China (grant numbers: 82060046).

## CONFLICT OF INTEREST STATEMENT

The authors declare that they have no conflict of interest.

## Data Availability

The data used to support the findings of this study are available from the corresponding author upon request.
